# Combating multidrug-resistant Helicobacter pylori with moenomycin A in combination with clarithromycin or metronidazole

**DOI:** 10.3389/fchem.2022.897578

**Published:** 2022-10-19

**Authors:** Yen-Yu Tseng, Jyh-Ming Liou, Wei-Chieh Cheng, Jing-Ting Hsu, Tsui-Ling Hsu, Ming-Shiang Wu, Chi-Huey Wong

**Affiliations:** ^1^ Genomics Research Center, Academia Sinica, Taipei, Taiwan; ^2^ Division of Gastroenterology, Department of Internal Medicine, National Taiwan University Hospital, Taipei, Taiwan; ^3^ Department of Chemistry, The Scripps Research Institute, La Jolla, CA, United States

**Keywords:** moenomycin, combination, Helicobacter, antibiotics, penicillin-binding protein knockout

## Abstract

Current treatment of *Helicobacter pylori* involves a triple therapy comprising one proton pump inhibitor and two other antibiotics; however, the outcomes are limited due to the existence of antibiotic resistant strains. We previously reported that moenomycin A, a cell-wall transglycosylase inhibitor, is highly active against multidrug-resistant *Helicobacter pylori*. Herein we show that combination of moenomycin A with the protein synthesis inhibitor clarithromycin or metronidazole can synergistically achieve almost 95% eradication of multidrug-resistant *Helicobacter pylori*. We also found that the moenomycin A-non-susceptible strains of *Helicobacter pylori* with deletion of transglycosylase exhibit moenomycin A hyposensitivity, faster growth and impaired biofilm formation compared to the parental strain. Overall, the combination of moenomycin A and clarithromycin or metronidazole to achieve a synergistic effect on different targets is a promising treatment for multidrug-resistant *Helicobacter pylori*.

## Introduction

Over half of the world’s population are infected with *Helicobacter pylori* (*H. pylori*), a microaerophilic Gram-negative bacterium. Separated from gastritis and peptic ulcer (Marshall et al., 1998), this bacterial infection was found to be the key risk factor of gastric cancer (Malfertheiner et al., 2012). Generally, the treatment of *H. pylori* infection involves a 1–2-week use of antibiotics including the combination of a proton pump inhibitor with two to three antibiotics such as clarithromycin, amoxicillin, metronidazole/tinidazole, tetracycline or levofloxacin to reduce acid production in the stomach (McColl, 2010; Malfertheiner et al., 2012; Liou et al., 2013a). However, about 20% of patients with *H. pylori* infection develop antibiotic resistance (Liou et al., 2013b; Megraud et al., 2013).

Moenomycin (Moe), known as flavomycin or bambermycin for fodder, is a mixture compounds (Moenomycins A, A12, C1, C3, and C4) which are separated from *Streptomyces* species (Wallhausser et al., 1965; Huber and Moenomycin, 1967; Huber and Nesemann, 1968). It was issued in the early 1960s as animal feedstock and was claimed to inhibit the transglycosylase (TGase) in Gram-positive bacteria to block the biosynthesis of peptidoglycan that forms bacterial cell wall (van Heijenoort et al., 1987; Chen et al., 2003; Halliday et al., 2006). Bacterial cell wall is mainly comprised of peptidoglycan which formed through two key reactions, transglycosylation and transpeptidation catalyzed respectively by the enzymes transglycosylase and transpeptidase which exist as separate domains of the penicillin binding protein (PBP). TGase catalyzes the transfer of the glycosyl moiety from a glycosyl donor to a hydroxyl group of lipid-II (van Heijenoort, 2007). TGase is an important antibiotic target since it is essential for bacteria and the polysaccharide backbone of the peptidoglycan is conserved in drug-resistant strains; therefore, antibiotics targeting the transglycosylation step may be difficult to develop resistance. On the other hand, the transpeptidase is extensively used for development of antibiotics such as β-lactam antibiotics and vancomycin. Due to the overuse or abuse of antibiotics that target the transpeptidase, some resistant strains have emerged.

There were reports on multi-drug resistant (MDR) *H. pylori* strains isolated from different gastrointestinal patients, but how these strains develop Moe A resistance was unknown (Orhan et al., 2005; Cheng et al., 2008; Salzberg et al., 2011; Tseng et al., 2014; Yuriy et al., 2014). So far, Moe A has been only used as animal feedstock and has never been developed for human use, probably due to its poor absorption. However, because of its extremely low absorption in the gastrointestinal tract with negligible toxicity, Moe A appears to be a suitable antibiotic for the treatment of patients infected with MDR *H. pylori* strains which are often found on the lining of the stomach. Based on our previous study *in vitro* (Tseng et al., 2014) and *in vivo* in animal models (data not shown), we found that MoeA has beneficial effect on multidrug-resistant *H. pylori* eradication *in vitro* but not *in vivo*. Hence, we tried to improve the therapeutic efficacy by combination therapy and we did see the synergistic effect *in vitro*. The *in vitro* method of detecting synergy is simple to manipulate, especially with Time-Kill, Checkerboard, and E test (White et al., 1996). Time-kill and Checkerboard methods are the most widely used techniques to analyze synergy. However, the Time-kill method of synergy test need to manipulate with the broth under microaerobic condition. Therefore, we selected convenient operation of Checkerboard methods with the agar under microaerobic condition to assess MoeA combination therapy for *H. pylori* eradication. Furthermore, using surface plasmon resonance analysis to compare the binding of moenomycin A with various truncated PBPs (Cheng et al., 2008), we previously found that the transmembrane domain of PBP is vital for moenomycin binding, particularly in the case of Gram-negative *H. pylori*. Hence, the purpose of this study was to study the efficacy of Moe A combination therapy on MDR *H. pylori* isolated from patients with gastrointestinal disease and to understand the role of PBP in Moe A-non-susceptible *H. pylori* strains.

## Materials and methods

### Chemicals

Moenomycin (>90%) was purified from FLAVOMYCIN 20 (Huvepharma, Antwerp, Belgium). Clarithromycin was purchased from K-mycin (Standard Chem and Pharm CO. Tainan, Taiwan) and metronidazole were obtained from FLAGYL (SHIONOGI and CO. Taipei, Taiwan).

### Patients and isolation of *H. pylori*

*H. pylori* strains were obtained from the stomach of patients infected by *H. pylori* who were treatment-naïve or treated with eradication therapies at National Taiwan University Hospital (NTUH), Taipei, Taiwan. Patients with a history of partial gastric resection were excluded. The study protocol was authenticated by the Institutional Review Boards of NTUH (Becton Dickinson and Co., Rutherford, New Jersey, United States). Bacterial plates were incubated at 37°C under microaerophilic conditions of 5% O_2_, 10% CO_2_ and 85% N_2_ for 4 days. All strain cultures were stored at −80°C until the need for investigation.

### Construction of the penicillin binding protein 1a knockout mutant

The gene encoding PBP1a with the upstream and downstream about 500 bp was amplified with the gDNA of the resistant strain by polymerase chain reaction (PCR). The primers of PBP1a KO were designed by Primer3Plus software: Upstream region (forward) CTC​GAG​TTC​TTT​CTT​GCA​AGA​TGT​GCC; Upstream region (reverse) GTA​TAT​CCA​GTG​ATT​TTT​TTC​TCC​ATC​TAC​ATG​GCT​ATA​GGG​ACT​TTA​ACA; Downstream region (forward) GGG​CGG​GGC​GTA​AAT​GTT​TTC​TAA​ATC​TTT​AGA​AGC​CCT; Downstream region (reverse) GGT​ACC​TTT​TAG​GGG​CGT​TAA​AGA​GTT​T. The PBP1a genes were truncated by multiplex PCR, and a chloramphenicol-resistant cassette about 660-bp codon was then cloned to replace the full length PBP1a gene. Furthermore, the DNA fragment was cloned into the pGEM-T easy vector (Promega, Madison, Wisconsin, United States). A chloramphenicol-resistant cassette with the upstream and downstream about 500 bp flanking region of PBP1a was cut off and ligated into the XhoI (New England Biolabs, Ipswich, Massachusett, United States) site and PknI (New England Biolabs, Ipswich, Massachusett, United States) of the shuttle vector pHel3 (plasmid pHel3 was a presentation from Rainer Haas, Universität München, Germany). This plasmid was electrotransformed into the resistant strain to generate truncating mutants. The DNA of the transformants was verified by PCR with external and internal primers and validated by DNA sequencing.

### Electrotransformation of *H. pylori*

*H. pylori* cells were transformed with plasmids (pHel3-PBP1a-Cmr) by electroporation. Briefly, the *H. pylori* culture on plates was scraped and suspended with ice-cold ultrapure water. The cells were collected by centrifugation, and the pellet was washed using ice-cold 10% glycerol (Sigma-Aldrich, Saint Louis, Missouri, United States) twice and resuspended with 10% glycerol. Plasmid DNA was mixed with cell suspension. The mixture was poured into a cooled electroporation cuvette (Bio-Rad, Hercules, California), and shocked with a single-pulse electroporation (2.5 kV, 25 mF, 200 Ω). The sample was incubated on serum plate for 24 h at 37°C. Finally, the bacteria were inoculated onto chloramphenicol selective media for 4 days.

### Checkerboard test

From the stock solutions, a serial dilution of each antibiotic was separated into each agar plate to obtain different concentrations of 0.125, 0.25, 0.5, 1, 2, 4, 8, 16, and 32 μg/ml for checkerboard analysis. The agar plates with one antibiotic and its combinations were placed by different concentrations. Thus, each of the agar plates presented a unique combination of concentrations of the two antibiotics. The plates were incubated for 3 days at 37°C. The fractional inhibitory concentration index (FIC I) was used to calculate the results. TheΣFractional inhibitory concentration (ΣFIC) values were counted as follows: ΣFIC = the fractional inhibitory concentration A (FIC A) + FIC B, where FIC A is the minimum inhibitory concentration (MIC) of antibiotic A in the combination/MIC of antibiotic A alone, and FIC B is the MIC of antibiotic B in the combination/MIC of antibiotic B alone. The combined effect is synergistic when ΣFIC I is ≤ 0.5. Additions was expressed by a FIC I from >0.5 to ≤1 and indifferent FIC I was >1 to ≤4 while antagonism is indicated as when the ΣFIC I is > 4 (Eliopoulos and Moellering, 1991; Salzberg et al., 2011).

### Biofilm quantification

Bacteria were cultured based on the growth curves; however, biofilm ring assays were manipulated by 24-well tissue culture plates. Bacterial cultures were used to inoculate in liquid medium with each well to the optical density (OD) at 600 nm (OD600) of 0.1 optical density unit (ODU). Each well was sucked dry and washed two times with phosphate-buffered saline (PBS, Sigma-Aldrich, Saint Louis, Missouri, United States). Plates were dried for 5 min at 37°C, and then biofilm rings were fixed with methanol. Gram’s crystal violet (Sigma-Aldrich, Saint Louis, Missouri, United States) solution was added to each well. The plates were incubated, crystal violet was removed, and each well was washed three times with ultrapure water. The plates were desiccated. Then, differentiation solution was used to solubilize the dye. Plates were incubated with differentiation solution, solubilized crystal violet solution was transferred to a cuvette, and the absorbance at 590 nm (OD590) was read. The data was shown with three biologically independent experiments.

### Scanning electron microscopy

Stock cultures were cultured in fresh Brain Heart Infusion Broth (BHI broth; Becton Dickinson and Co., Rutherford, New Jersey, United States) in plates. Biofilms were captured on coverslips and analyzed by scanning electron microscopy. Briefly, samples were fixed. After fixation, samples were washed three times with 0.05 M sodium cacodylate buffer. The samples were mounted onto carbon stubs, vacuumed sputter-coating with gold, observed with SEM. A Hitachi S-2400 N SEM (Hitachi High-Technologyies, Tokyo, Japan) was used to acquire all images.

### Growth inhibition

Cultures of parental and knockout strains were diluted with fresh broth media. Moe A was added at 2-fold serial dilution from the highest concentration (0–16 µg/mL) directly to the culture tubes and the turbidity was monitored 4 days later by reading OD600.

### Motility assay

Cultures of parental and knockout strains were diluted with fresh broth media, followed by incubation for 1–5 days and the turbidity was recorded by OD600.

### Detection of PBPs from membrane

To detect PBPs with BOCILLIN FL (Thermo Fisher Scientific, Waltham, Massachusett, United States) labeling reagent, *H. pylori* cells were cultured on plates. The bacteria were collected by centrifugation, then washed with PBS, resuspended in the same buffer, and disrupted by sonication. The cell lysates were centrifuged. The supernatant fractions were harvested and centrifuged. The pellets were harvested, washed, and resuspended. The suspensions were utilized for membrane preparations and fluorescent binding assays with BOCILLIN FL. The protein concentrations on the membrane were quantified by using the Bradford protein assay (Bio-Rad Laboratories Hercules, California, United States). For detection of PBPs, each membrane preparation (≈300 μg of protein) was subjected to SDS-PAGE (Thermo Fisher Scientific, Waltham, Massachusett, United States) and proteins were blotted onto PVDF membrane (Merck Millipore Burlington, Massachusetts, United States). Non-specific binding was blocked in 5% blocking buffer for 60 min, and membranes were incubated with 10 μM BOCILLIN FL at 37°C for 60 min. To visualize the labeled PBPs, the PVDF was visualized under UV light (290 nm).

## Results

**FIGURE 1:**
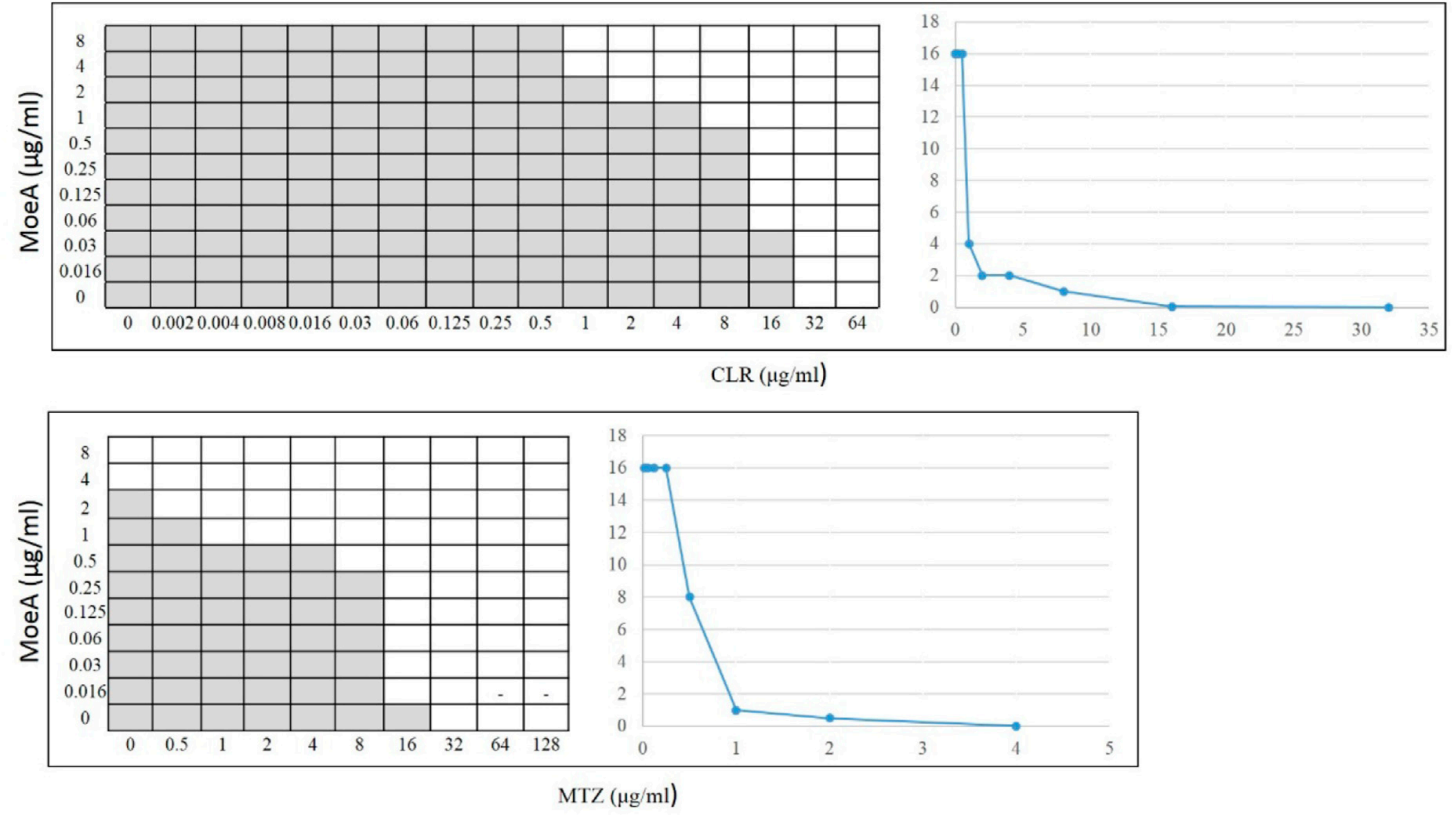
Checkerboard assays and isobolograms for assessment of synergism in the (MoeA + CLR) combination against No. 2 *H. pylori* strain or the (MoeA + MTZ) combination against No. 3 *H. pylori* strain. The interpretation of the best (MoeA + CLR) or (MoeA + MTZ) combination was based on synergism (FIC I ≤ 0.5), antagonism (FIC I ≥ 4.0), and additive effect (FIC I > 0.5–4.0). The grey zone represents bacterial viability, and the white zone represents bacterial mortality in the presence of (Moe A+ CLR) or (Moe A+ MTZ). On the right, the isobolograms showed the synergistic curve. The *x* axis of the isobologram is the dose of CLR or MTZ and the *y* axis is the dose of Moe A.

Our previous report indicated that Moe A was effective against MDR *H. pylori* strains *in vitro* but the *in vivo* effect in animal models was not as significant as the *in vitro* effect. To further evaluate the efficacy of Moe A combination therapy, the effect of Moe A in combination with clarithromycin (CLR) was evaluated against MDR strains to determine the susceptibility. Table 1 shows the best combination of Moe A and CLR with FIC I. Synergism was found in 21 of 31 strains, additive effect was found in 9 of 31 strains and indifferent effect was displayed in 1 of 31 strains (FIC I = 1.063). Antagonism was not observed. The MICs of CLR for MDR strains decreased when tested in combination with Moe A with concentrations ranging from 0.001 to 11.167 μg/ml. Particularly, the best FIC I was observed for No. Three strain with MIC of 0.019 μg/ml, corresponding to a 66- and a 266-fold reduction of MIC for Moe A and CLR, respectively. In addition, the effect of Moe A in combination with metronidazole (MTZ) was evaluated against MDR strains to determine the susceptibility (Table 2). Table 2 shows the best combinations of Moe A and MTZ with FIC I. Synergism was observed in 21 of 32 strains, additive effect was observed in 10 of 32 strains and indifferent effect was displayed in 1 of 32 strains (FIC I = 1.063). Antagonism was not observed. The MICs of MTZ and Moe A combination against MDR strains were lower than the MICs of Moe A with concentrations ranging from 1.25 to 21.333 μg/ml. Especially, the best FIC I was observed for No. Two strain with MIC of 0.229 μg/ml, corresponding to a seven- and a 14-fold reduction of MIC for Moe A and MTZ, respectively. In addition, Figure 1 shows the checkerboard assays and the isobolograms obtained from different concentrations of Moe A and CLR against No. Two strain, the (MTZ + CLR)-resistant strain or from different concentrations of Moe A and MTZ against No. Three strain, the (MTZ + CLR)-resistant strain.

**TABLE 1:** FIC I of MoeA and CLR against multidrug resistant *H. pylori* strains.

	MIC (ug/mL)		Best combination MIC (ug/mL)		FIC I		Σ FIC		
							Synergy	Additive	Indifferent
No. of strains	MoeA	CLR	MoeA	CLR	MoeA	CLR	≤0.5	0.5-<1	1-<4
MTZ + CLR resistant									
No. 2	6.333	23.333	1.343	6.003	0.385	0.107			
No. 3	2	16	0.03	0.06	0.015	0.004	0.019		
No. 4	1.667	14	0.26	0.062	0.213	0.011	0.225		
No. 8	8	128	1	1	0.125	0.008	0.132		
No. 9	9	32.03	1	0.501	0.281	0.024	0.306		
No. 10	1	64.02	0.542	0.751	0.542	0.018		0.559	
No. 11	2	90.667	0.343	0.067	0.218	0.002	0.22		
No. 12	1	0.123	0.197	0.052	0.197	0.356		0.552	
No. 14	7	21.708	0.51	0.063	0.26	0.163	0.423		
No. 15	4	0.182	0.833	0.022	0.354	0.146	0.4997		
No. 16	1.333	0.0227	0.093	0.001	0.073	0.433		0.506	
No. 17	0.5	42.707	0.135	0.336	0.51	0.047		0.557	
No. 18	1	66.667	0.135	11.167	0.135	0.19	0.325		
No. 20	10	33	2	4.002	0.313	0.064	0.376		
No. 21	9	48	1.015	0.254	0.07	0.08	0.078		
No. 22	4.667	48.017	1.343	0.337	0.501	0.014		0.515	
No. 23	1.167	26.687	0.343	0.354	0.427	0.018	0.444		
No. 25	0.667	5.52	0.103	0.349	0.123	0.198	0.322		
No. 28	2.667	26.687	1.353	5.347	0.51	0.095		0.605	
No. 29	16	8	6.667	1.005	0.417	0.126		0.542	
CLR + LEV resistant									
No. 5	0.583	13.333	0.2183	0.167	0.353	0.016	0.369		
No. 6	1.667	45.5	0.667	2.834	0.417	0.044	0.461		
No. 7	10	8.03	2.5	0.003	0.531	0.017		0.548	
No. 26	1	13.353	0.343	5.335	0.343	0.178		0.521	
No. 31	1.667	13.353	0.113	0.049	0.08	0.277	0.358		
No. 32	1	24.02	1.01	5.335	1.01	0.095			1.105
No. 33	2.333	29.333	0.395	10.692	0.213	0.169	0.382		
No. 34	7	25.333	0.26	0.096	0.174	0.095	0.197		
MTZ + CLR + TET resistant									
No. 19	1.333	8.167	0.375	1.359	0.292	0.207	0.499		
No. 24	6.333	8.667	0.26	0.667	0.099	0.189	0.2875		
No. 27	3.333	16.02	1.5	0.678	0.417	0.2083		0.625	

**TABLE 2:** FIC I of MoeA and MTZ against multidrug resistant *H. pylori* strains.

No. of strains	MIC (ug/mL)	Best combination MIC (ug/mL)		FIC I			Σ FIC		
							Synergy	Additive	Indifferent
	MoeA	MTZ	MoeA	≤0.5	0.5-<1	1-<4	≤0.5	0.5-<1	1-<4
MTZ + CLR resistant									
No. 2	4.333	26.67	0.583	1.833	0.167	0.063			0.229
No. 3	2.5	32	0.75	4.5	0.375	0.141		0.516	
No. 4	2.333	26.67	0.417	3	0.208	0.099	0.307		
No. 8	4	32	1	2	0.25	0.063	0.313		
No. 9	2.5	32	0.75	1.25	0.375	0.039	0.414		
No. 10	1.667	106.7	0.353	12.17	0.26	0.089	0.349		
No. 11	1.333	96	0.672	5.667	0.669	0.042		0.711	
No. 12	1	106.7	0.255	11	0.255	0.053	0.307		
No. 13	2.667	14.67	0.353	4.333	0.174	0.333		0.508	
No. 14	2.333	93.33	0.672	3.167	0.255	0.063	0.318		
No. 15	5.333	53.33	0.692	16.17	0.171	0.255	0.426		
No. 16	3.333	53.33	0.667	13.5	0.375	0.214		0.589	
No. 17	0.917	18.67	0.045	2	0.123	0.146	0.269		
No. 18	1.333	64	0.505	5.833	0.339	0.091	0.43		
No. 20	6.667	42.67	1.667	8	0.25	0.208	0.458		
No. 21	3	32	1.5	4.5	0.5	0.141		0.641	
No. 22	3.667	42.67	1.01	5.833	0.422	0.177		0.599	
No. 23	8	64	1	4.333	0.188	0.068	0.255		
No. 25	1.333	64	0.5	1.667	0.417	0.026	0.443		
No. 28	1.667	42.67	0.422	16	0.253	0.417		0.699	
No. 29	16	170.7	8	11.17	0.5	0.087		0.587	
CLR + LEV resistant									
No. 5	21.12	18	0.515	1.5	0.185	0.156	0.342		
No. 6	4.333	24	0.51	2.333	0.093	0.271	0.364		
No. 7	2.5	4	1	1.25	0.75	0.313			1.063
No. 26	1.667	5.333	0.192	2.667	0.106	0.5		0.606	
No. 31	0.833	97.33	0.177	3.333	0.183	0.214	0.396		
No. 32	2.333	12	0.667	1.667	0.375	0.298		0.583	
No. 33	3.333	53.333	0.677	12	0.172	0.271	0.443		
No. 34	2.667	53.333	0.667	5	0.292	0.102	0.396		
MTZ + CLR + TET resistant									
No. 19	2.667	74.67	0.51	16.17	0.213	0.214	0.437		
No. 24	2	42.67	0.583	3	0.333	0.911	0.424		
No. 27	3.333	64	0.339	21.33	0.126	0.333	0.46		

**FIGURE 2:**
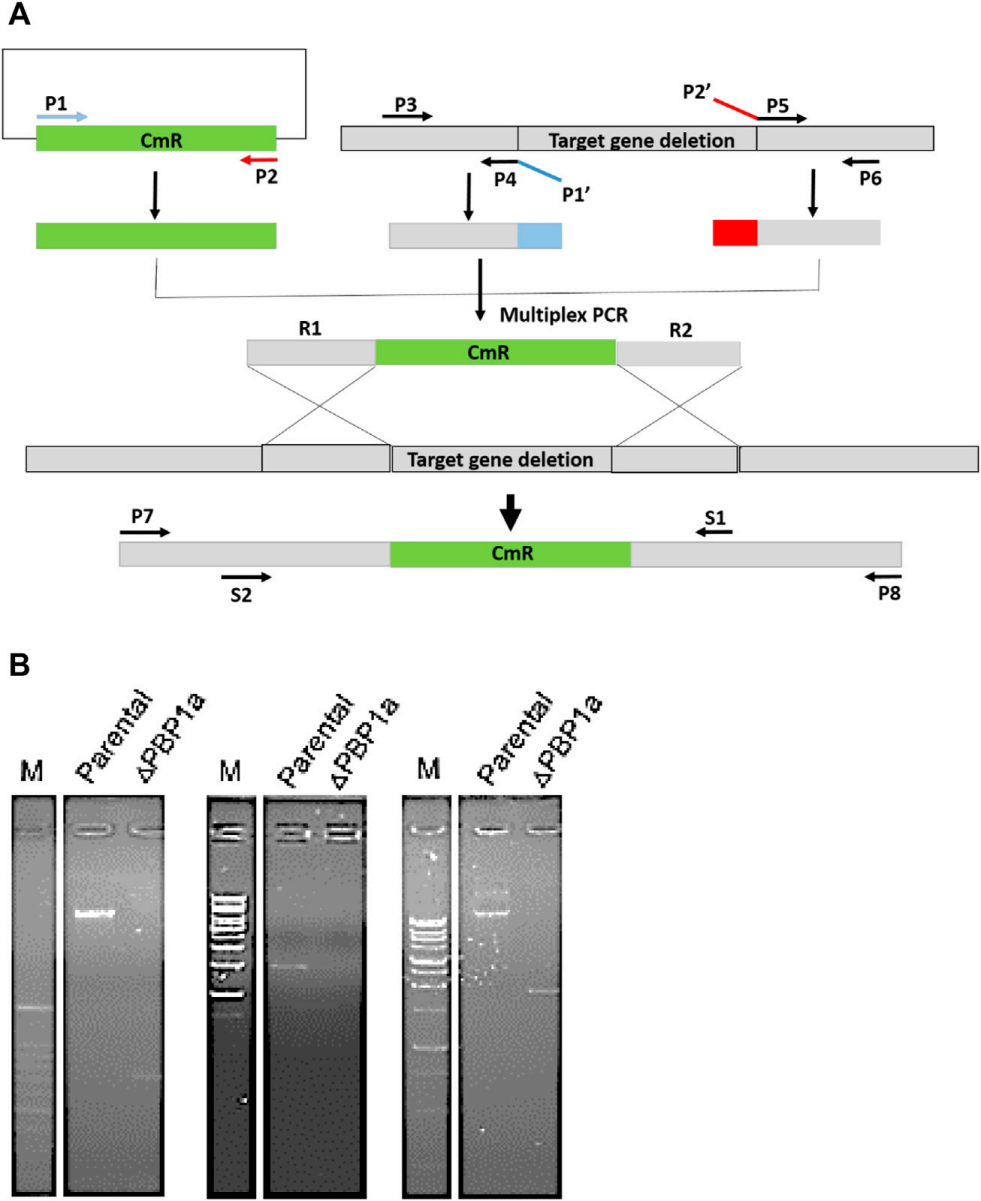
**(A)** The flowchart of gene truncation in *H. pylori*. The fragments of upstream and downstream were amplified with primers P3 and P4 and with P5 and P6, respectively. The CmR selective marker fragments was amplified with primers P1 and P2. Primers P1′ and P2′ were devised for generic linkers (shown in blue and red). The fragments of upstream and downstream were linked to the CmR fragments by using nested primers (S1 and S2). **(B)** Long range PCR analysis of genomic DNA from parental and ΔPBP1a before and after pHeL3 transfection was performed to confirm the integration (660 bp), the upstream flank (1,269 bp) and the downstream flank (1,953 bp), respectively.

**FIGURE 3:**
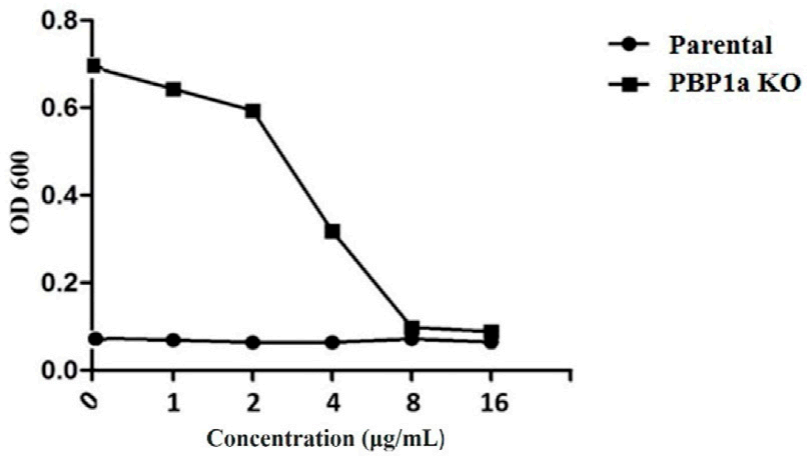
Comparison of sensitivity between parental and PBP1a KO strains for serial dilution antibiotic susceptibility testing. MIC of the parental -type and PBP1a KO strain in response to 0, 1, 2, 4, 8, 16 μg/ml MoeA. The results of at least three independent experiments are calculated.

At present, the mechanism of Moe A resistance to *H. pylori* is not completely understood. Two Moe A-resistant *H. pylori* strains were isolated from patients of NTUH. The strains showed a high degree of similarity with ATCC43504 through PBP1a detection and identification. To clarify if PBP1a in these strains interacts with Moe A, PBP1a deletion mutants were constructed and confirmed with sequencing and functional assays as below and Cmr colonies were isolated ​(Figure 2A). The DNA was amplified with the primers P7/S1 and S2/P8 annealing to PBP1a of the parental strain to produce a 1,269 bp and a 1,953 bp sequence, separately ​(Figure 2B). A 660 bp product was amplified and sequenced from truncated strains. The results showed upstream and downstream genes of PBP1a as well as cmr gene instead of PBP1a, indicating success of the assembly (data not shown). To assess whether the PBP1a gene was involved in Moe A resistance, the PBP1a of Moe A-non-susceptible strain was knocked out. As shown in Figure 3, the PBP1a deletion strain grew in media containing 8 µg/ml of Moe A (MIC = 2 µg/ml). These results demonstrate that the genetic alteration of PBP1a gene may cause Moe A resistance. Because the parental strain showed a high-level Moe A resistance (MIC = 8 µg/ml) with genetic alteration in the PBP1a gene. This finding raised the question that the high-level Moe A resistance might be caused by PBP1a inactivation.

**FIGURE 4:**
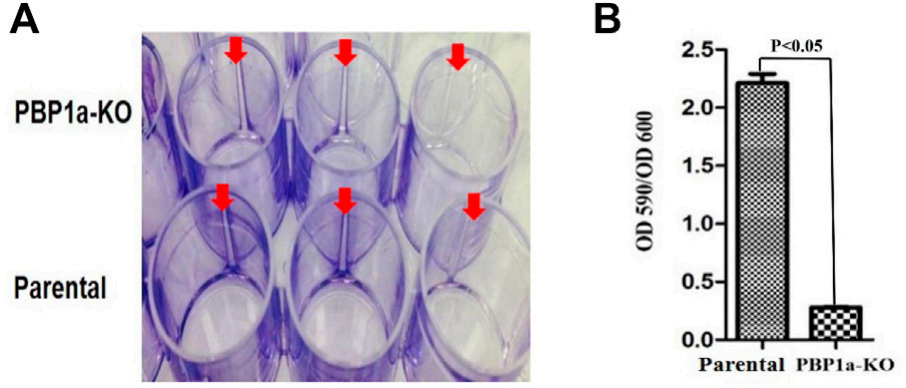
Effect of PBP1a KO on biofilm formation in a-non-susceptible clinical *H. pylori* strain. **(A)**, Qualitative biofilm ring (arrow) in 24-well Corning Costar cell culture plates after crystal violet staining. Row A and B represent PBP1a KO strain and the parental strain respectively. **(B)**, Quantitative biofilm formation in PBP1a KO strain and the parental strain. Standard deviation of optical densities (OD 590/OD 590) was from three independent experiments. Statistically significant difference (*p* < 0.05) was calculated by the *t*-test.

**FIGURE 5:**
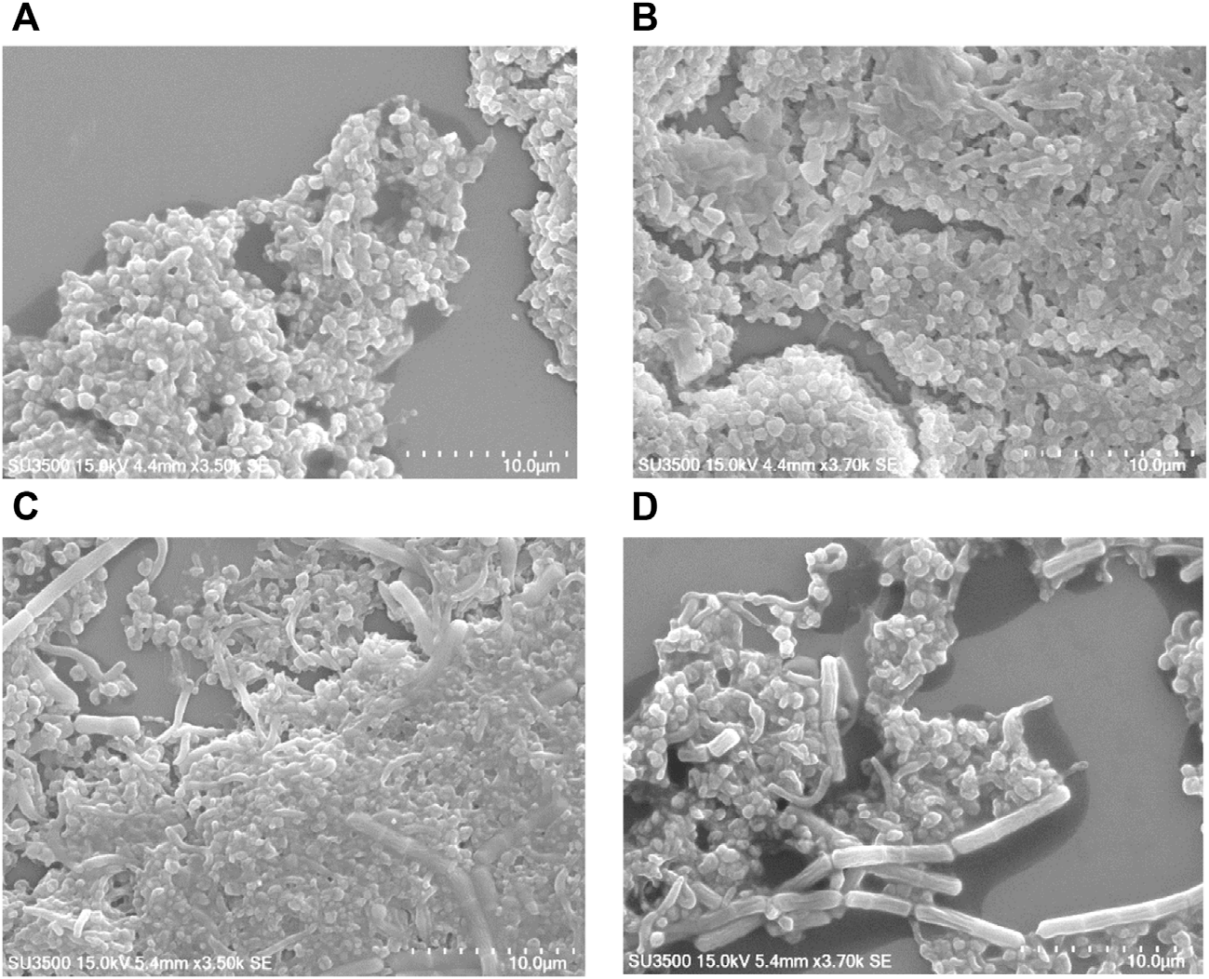
Effect of PBP1a deletion on the biofilm formation of a non-susceptible clinical *H. pylori* strain under SEM. Scanning electron micrographs were taken to show the structural differences in biofilm architecture of parental strain vs. PBP1a deletion strain. Bacteria were grown on cell culture-treated coverslips for 24 h (scale bars, 10 µm). SEM micrograph of the biofilm formation of parental strain **(A)** at magnification 3.5 k×, voltage 15.0 kV, working distance 4.4 mm, with 10 μm scale bar. **(B)** At magnification 3.7.0 k×, voltage 15.0 kV, working distance 4.4 mm, with 10 μm scale bar. **(C)** SEM micrograph of the biofilm formation of PBP1a KO strain at magnification 3.5 k×, voltage 15.0 kV, working distance 5.4 mm, with 10 μm scale bar. **(D)** At magnification 3.7 k×, voltage 15.0 kV, working distance 5.4 mm, with 10 μm scale.

In many bacteria, PBP contributes to biofilm forming. We evaluated the capability of *H. pylori* to form biofilm rings with an Improved Crystal Violet Assay, performed in 24-well polystyrene microtiter plates with measurements at 72 h. We observed that the Moe A-non-susceptible PBP1a deletion strain formed the weak biofilm ring (Figures 4A,B). In contrast, the parental strain formed moderate to the strong biofilm ring. Besides, we also applied SEM to gain more insight into the biofilm structure. We could observe mighty biofilm formation and countless adherent bacteria that contained mainly coccoid forms along with some bacilli. However, biofilm and adherent bacteria were less monitored for the PBP1a deletion strain (Figure 5). Interestingly, Rod-shaped cells were mostly visible in the PBP1a deletion strain. Despite these differences, the SEM data suggest that PBP1a is sophisticatedly involved in the biofilm formation of *H. pylori*.

**FIGURE 6:**
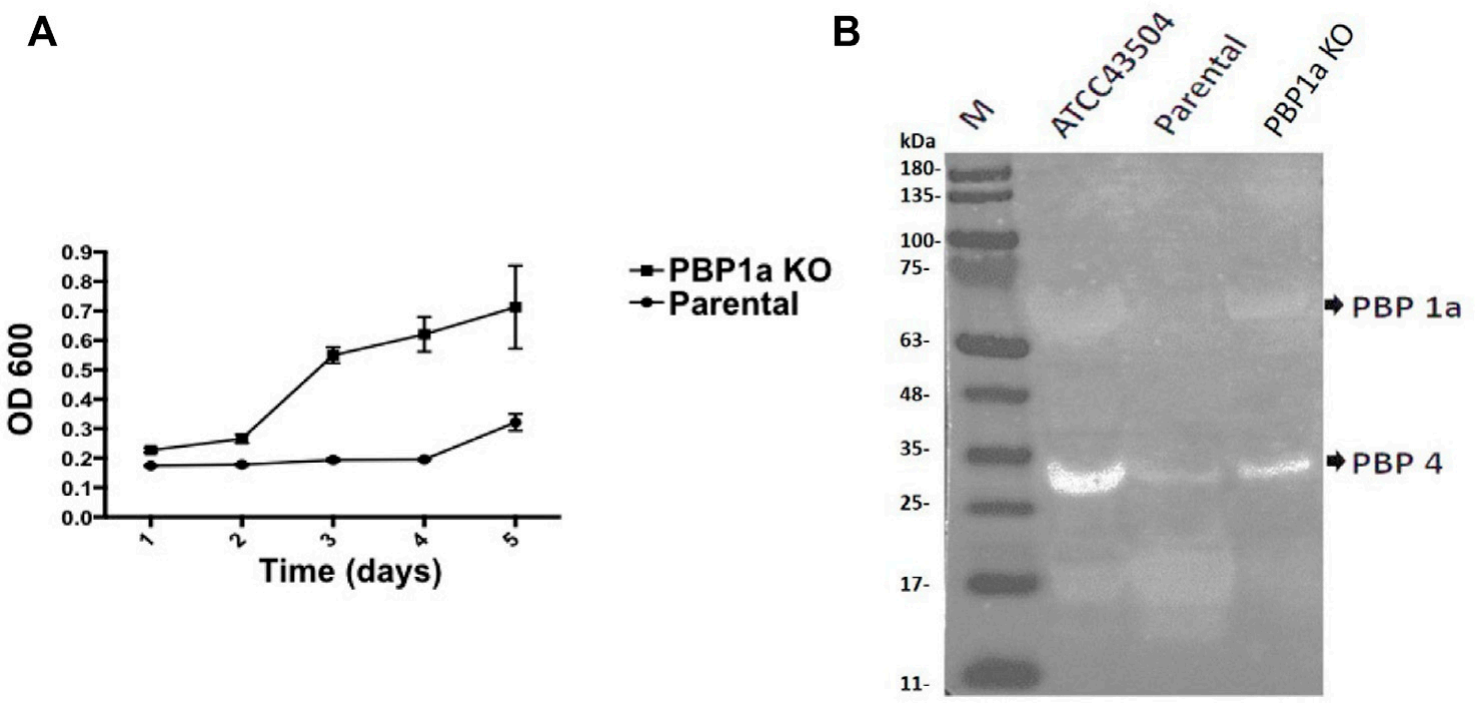
**(A)** Effect of PBP1a deletion on the growth rate of a non-susceptible clinical *H. pylori* strain. Growth curves of a non-susceptible clinical *H. pylori* strain (circles), and its PBP1a KO strain (squares). Each data point represents the mean and standard deviation (bars) of OD from triplicate wells. **(B)** PBP profiling of *H. pylori*. Extracted membrane labeled BOCILLIN FL were visualized under UV light (290 nm); lane 1, *H. pylori* ATCC 43504; lane 2, a non-susceptible clinical *H. pylori* strain; lane 3, PBP1a KO of the non-susceptible clinical *H. pylori* strain.

The growth rate of parental strain was then analyzed by turbidimetry and compared to the PBP1a deletion strain. (Figure 6A), and it was shown that the PBP1a deletion strain exhibited a faster growth rate than the parental strain. The bacterial mass of the PBP1a deletion strain increased exponentially from day 2 to day 5 but the bacterial mass of Moe A-non-susceptible strains remained in a steady state until day 4. The differences in growth between the PBP1a deletion strain and the parental strain could be interpreted as a direct consequence of PBP1a deletion or mutation in elevating the MICs of Moe A. To determine whether other PBPs were involved in the growth rate, the PBP of parental strain was analyzed by BOCILLIN FL and compared to the PBP1a deletion strain. (Figure 6B). Two PBPs were identified in *H. pylori*. We refer to the PBP with 55–70 kDa as PBP 1a and the PBP with 32 kDa as PBP 4. The parental strain exhibited a significant loss of PBP four signal compared to the PBP1a deletion strain. The PBP4 difference between the deletion of PBP1a could result in the differences in growth.

## Discussion

Our findings in this study support the proposition that combination of Moe A with one of other antibiotics at present utilized for the treatment of *H. pylori* infection may eradicate *H. pylori* and its MDR strains. The eradication efficacy using the combination of Moe A and CLR was like the combination of Moe A and MTZ. Treatment of (MTZ + CLR)-resistant and (CLR + LEV)-resistant strains with Moe A in combination with CLR was shown to have more than 64% synergistic effect and 32% additive effect from the two antibiotics, and the combination effect of Moe A and CLR on (MTZ + CLR + TET)-resistant strains was 100% synergistic. If there is indeed a greater benefit for the Moe A combination therapy in these MDR strains, the synergistic effect is the mode of action (Caesar and Cech, 2019). Besides, the combination of Moe A and MTZ against the (MTZ + CLR)-resistant or the (CLR + LEV)-resistant strains was shown to have more than 62% synergistic effect and 34% additive effect. However, the Moe A and MTZ combination therapy against the (MTZ + CLR + TET)-resistant strains was also found to have 100% additive effect. Since CLR and TET are both protein synthesis inhibitors, the additive effect perhaps was more significant than the synergistic effect for the (MTZ + CLR + TET)-resistant strains (Gasparetto et al., 2012; Bollenbach, 2015).

**FIGURE 7:**
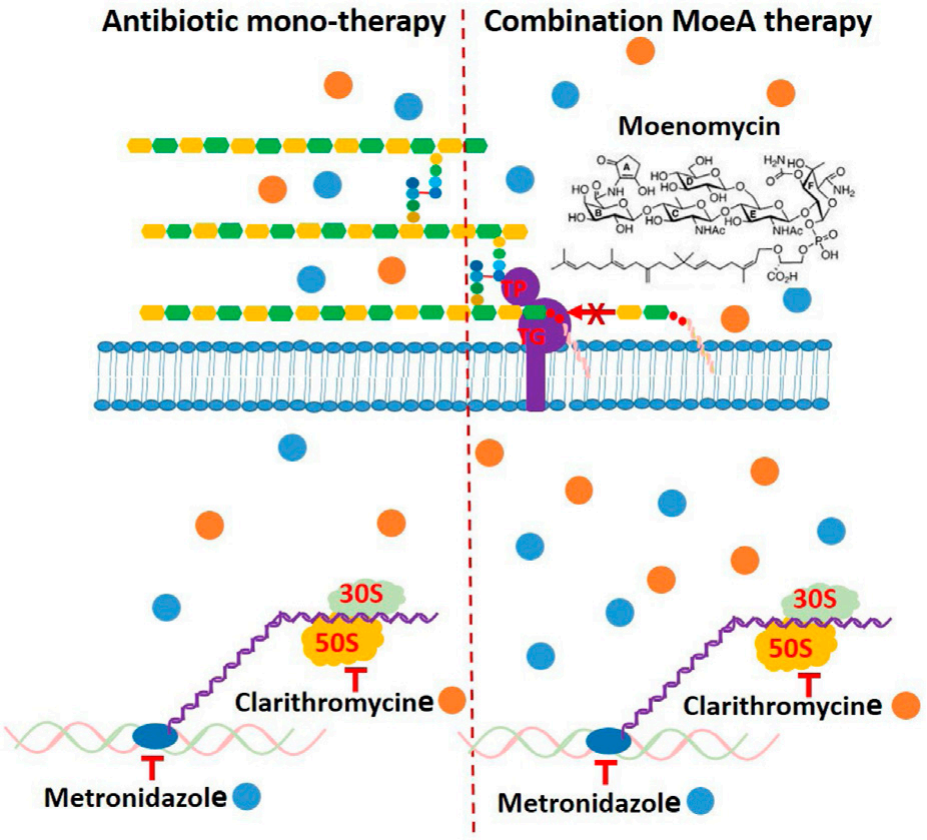
Schematic presentation of the mechanism of antibiotic monotherapy or combination Moe A therapy and possible binding of cytosolic proteins and genes, which might be a reason for enhancing antimicrobial activity.

Based on our previous data (Caesar and Cech, 2019), the incidence rate of Moe A-resistance to one, two, three, four, and five drugs were 16.7%, 27.3%, 29.4%, 20%, and 50%, respectively. Previous reports suggest that combination of antibiotics for the treatment of MDR *H. pylori* strains may be beneficial if the efficacy is ≥ 90% (Bergamaschi et al., 2007). Indeed, the treatment of *H. pylori* resistant to (MTZ + CLR), or (CLR + LEV), or (MTZ + CLR + TET) with (Moe A+ MTZ) or (Moe A+ CLR) was found to increase the effect of eradication (almost 90% vs. 27.3%) in this study. In our combination study, Moe A may disrupt the synthesis of peptidoglycan and affect cell-wall permeability, thus allowing CLR or MTZ to reach ribosomes or nucleus more easily and increasing their antibacterial activity by synergistic or additive effect (Figure 7).

**FIGURE 8:**
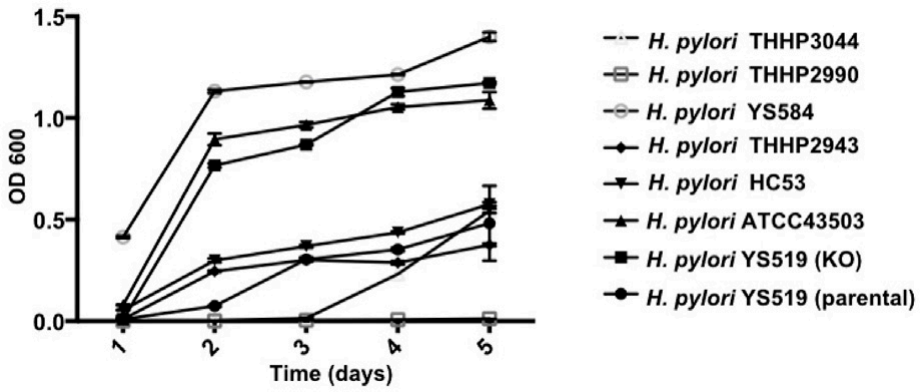
Growth curves of *H. pylori* ATCC43054 (Standard strain), *H. pylori* YS519 (parental), *H. pylori* YS519 (PBP1a KO), and other clinical Moe A-non-susceptible *H. pylori* strains. Growth curves were started at an OD600 of 0.05 in BHI medium, in triplicate.

Since PBP1a is a target for anti-bacterial drug design, we also investigated in this study the impact of PBP1a deletions on phenotypes, including susceptibility, cell morphology and biofilm formation. We sequenced the PBP1a of clinical strains and showed a high degree of similarity with ATCC 43504, and we reported for the first-time construction, verification, and characterization of PBP1a deletion strain using a pHel3 shuttle vector and found that deletion of PBP 1a had a high impact on bacterial growth, making cells to maintain in the rod forms. We believe this finding is significant and further research are needed to understand this interesting observation. The impaired biofilm formation with PBP1a deletion is consistent with the observation of PBP mutants in *E. coli* (Kumar et al., 2012), which exhibited significantly abated biofilm formation and motility. Biofilms are used to protect bacteria from antibiotic action and reduced antibiotic susceptibility (Stewart, 2002). In contrast, *H. pylori* with PBP1a KO are hyposensitive (Figure 3). Besides, the growth of *H. pylori* isolated from clinical patients has an elongating lag phase with maximum growth on day 5, while the standard strain (ATCC43504) with PBP1a deletion had maximum growth on day 4 (Figure 8). It appeared that the growth patterns of Moe A-non-susceptible strains isolated from clinical patients had longer lag phases compared to the standard strain. In *E. coli*, PBP1a mutants were shown that growth is more slowly, and viability also is lower than the wild-type and cause cells to become more sensitive to stress. Accordingly, AmpC, may aid resistance to β-lactam antibiotics (Pepper et al., 2006). However, based on our MIC experiments, the PBP1a deletion strain was less susceptible to the antibiotic than the parental strain. Although antibiotic sensitivity has been reported in *E. coli* PBP mutants, in *P. aeruginosa*, PBP mutants were antibiotic tolerant (Moya et al., 2009). Furthermore, inactivation of the *E. coli* dacB ortholog encoding PBP4 (Kishida et al., 2006; Ghosh et al., 2008) was demonstrated to be resistant to β-lactam antibiotics (Avison et al., 2001), and in general, the coccoid forms comprised high fatty acid and cholesterol showed antibiotic resistance (Kadkhodaei et al., 2020). Nevertheless, electron microscopy revealed that the cells with PBP1a deletion were rod-forms (Figures 5C,D), and this markedly altered susceptibility further emphasizes the importance of PBP1a in *H. pylori* physiology. Generally, the rod-form is viable, culturable and more susceptible compared with the coccoid form. In contrast to the current understanding, deletion of PBP1a induced a faster growth rate and produced more biomass, emphasizing the importance of PBP1a in antibiotic susceptibility. It is noted that PBP four of the PBP1a deletion strain appears to be overproduced compared to the parental strain (Figure 6B) and may contribute to the formation of the spiral form of *H. pylori* (Tuomanen, 1986). Moreover, it was also demonstrated that *E. coli* could grow at the biggest growth rate with fewer PBPs (Driehuis and Wouters, 1987; Guinote et al., 2011). BolA is a transcription factor to suppress the expression of the mreB gene. The alleviation of bacterial growth and survival can be considered to the overexpression of the bolA gene in *E. coli*, especially in the exponential growth phase. These data suggest that there is a significant regulation is between the peptidoglycan polymerization and cell growth. Overall, the effect of PBP1a on cell growth and cell shape could regulate the transcription of the gene involved in Moe A non-susceptibility.

## Conclusion

In this research, our study provides an encouraging strategy that combination of Moe A with other antibiotics currently used in gastrointestinal therapy is potentially useful for the treatment of infection caused by MDR *H. pylori* strains. The study of PBP1a deletion on Moe A-non-susceptible strains also provided useful information for the future design of better therapies against Moe A-non-susceptible *H. pylori* infection.

## Data Availability

The original contributions presented in the study are included in the article/supplementary material, further inquiries can be directed to the corresponding authors.
